# Remarkable Petition for Insane Asylum Investigation

**Published:** 1879-07

**Authors:** 


### Editorial.

### Remarkable Petition for Insane Asylum Investigation.

The *Albany Argus* of a recent date gives an editorial summary a very extraordinary report of the Committee on Public Health of the State Senate, made just before adjournment. To that committee was referred a petition alleging abuses in the State Asylum for the Insane. The committee were directed to inquire whether, from the averments of the petitioners or from any probable evidence they could furnish, warrantable ground existed for ordering a detailed investigation. The members of the committee put themselves in communication with the signers of the petition, and requested them to communicate facts within their knowledge. The Superintendents of State Asylums and the Commissioners in Lunacy were also summoned.

The result was very remarkable. More than half the petitioners repudiated their signatures altogether. President Barnard and Prof. Draper of New York were among those who declared that they never signed their names and that they were put on the petition by fraud. In one instance it was found that the signatures of the members of a large mercantile firm and twenty-one employees had been cut from a testimonial to which they were signed and pasted to the petition without the owners'. knowledge. The small number who admitted signing the petition had almost no knowledge of the asylums. They had not even visited them. The principal complainant, William A. Hammond of New York, had been in two of the asylums at intervals of eight and thirteen years; in others never. The committee therefore could get no facts whatever sustaining the charges. One or two witnesses showed great personal antipathy to certain superintendents of asylums, and that is all. The *Argus* says:

"The report, after, exhibiting facts like these, sums up the conclusion of the whole movement in very strong words. The committee declare the petition to be unsupported by a scintilla of evidence; they declared the action and evidence of the few who come to the front to be *an outrage on the honor of a decent and respected profession;* they declare the charges, so far as they can be made stand up in sight, to be not only unproved but disproved; they declare the process of getting signatures to be *an abuse of the right of petition which is beyond characterization. Malice, misrepresentation and ignorance, aye,* FALSEHOOD, *are made the bottom motives of the whole movement, by the report.* The report is very long and very elaborate. It will be one of the ablest legal and literary documents emanating from any legislative body of our time. The public interest in it is not small, and, therefore, we have here indicated its scope and character. The medical interest in the report will be universal. Every medical society in the United States should have a copy. As many doctors as can get copies should get them. The medical publications should present the report to their readers."—*Buffalo Express.*

The report of course does not pretend to show the present asylum management is all it should be. It goes no further than to say that there is no evidence whatever of abuses. At the same time it throws a painful light upon the value of petitions and upon the character and motives of the chief instigators of the raid upon insane asylums which has been going on in the legislature the past season. No wonder they ask for female Assistant Physicians in the female Wards of Insane Asylums and investigation of Asylums. This report of the state senate is conclusive official condemnation of the men, their motives and their measures.

